# Prediction of opioid dose in cancer pain patients using genetic profiling: not yet an option with support vector machine learning

**DOI:** 10.1186/s13104-018-3194-z

**Published:** 2018-01-27

**Authors:** Anne Estrup Olesen, Debbie Grønlund, Mikkel Gram, Frank Skorpen, Asbjørn Mohr Drewes, Pål Klepstad

**Affiliations:** 1 Mech-Sense, Department of Gastroenterology and Hepatology, Aalborg University Hospital, Aalborg, Denmark; 2 Department of Drug Design and Pharmacology, Faculty of Health and Medical Sciences, University of Copenhagen, Copenhagen, Denmark; 3 Department of Clinical Medicine, Aalborg University, Aalborg, Denmark; 4 Department of Clinical and Molecular Medicine, Norwegian University of Science and Technology, Trondheim, Norway; 5 Department of Cancer Research and Molecular Medicine, European Palliative Care Research Centre, Norwegian University of Science and Technology (NTNU), Trondheim, Norway; 6 Department of Circulation and Medical Imaging, Norwegian University of Science and Technology (NTNU), Trondheim, Norway; 7 Department of Anesthesiology and Intensive Care Medicine, St Olavs Hospital, Trondheim University Hospital, Trondheim, Norway

**Keywords:** SNPs, Cancer pain, Support vector machine, Genetics

## Abstract

**Objective:**

Use of opioids for pain management has increased over the past decade; however, inadequate analgesic response is common. Genetic variability may be related to opioid efficacy, but due to the many possible combinations and variables, statistical computations may be difficult. This study investigated whether data processing with support vector machine learning could predict required opioid dose in cancer pain patients, using genetic profiling. Eighteen single nucleotide polymorphisms (SNPs) within the µ and δ opioid receptor genes and the catechol-*O*-methyltransferase gene were selected for analysis.

**Results:**

Data from 1237 cancer pain patients were included in the analysis. Support vector machine learning did not find any associations between the assessed SNPs and opioid dose in cancer pain patients, and hence, did not provide additional information regarding prediction of required opioid dose using genetic profiling.

## Introduction

Opioids are the basis in treatment of severe pain of both benign and malignant origin. Unfortunately, the clinical use is limited by large inter-individual differences in analgesic responses, and insufficient treatment is often seen. Unacceptable side effects may also appear, potentially reducing quality of life. Thus, identification of biomarkers that can predict the appropriate opioid type and dose for the individual patient is highly warranted. Currently, there is no well-validated objective means of identifying patients likely to experience adequate opioid analgesia, and quantitative sensory testing as well as clinical biomarkers have been applied with low success [1–3].

Various factors such as gender, age, and genetic variation may influence the analgesic response to opioids. Several single nucleotide polymorphisms (SNPs) in different candidate genes have been demonstrated to be associated with altered pain sensitivity and analgesic response [4]. The genetic variations can influence the pharmacokinetics and/or pharmacodynamics of opioids and potentially the effect. A large study, the European Pharmacogenetic Opioid Study (EPOS) included 2294 cancer pain patients and investigated the influence of genetic variability on multiple pain-related outcomes and required opioid dose [5, 6]. No significant associations were found between 112 SNPs in 25 candidate genes and opioid dose, thus the findings did not support the use of genetics profiling to guide opioid treatment. However, statistical analysis including multiple testing of several factors may be a limitation. In contrast, machine learning can include many factors in a single analysis, limiting the risk of erroneous false-positive results [7]. Support vector machine (SVM) is a data driven method, which enables detection of subtle patterns in complex datasets, which are only visible when assessing multiple variables at once. This could be the case for genetic data, where certain combinations of genes could determine the effect.

The objective of the present study was to use SVM analysis of various SNPs to predict the required opioid dose in cancer pain patients.

## Main text

### Methods

#### Study design and patient samples

Data from the EPOS study was used for analysis [6]. In brief, the study included patients from 17 centres in 11 European countries. Inclusion criteria were: age > 18 years; diagnosed with a malignant disease; using opioids for moderate to severe pain; treated with regular oral, subcutaneous, transdermal or intravenous opioids (morphine, methadone, fentanyl, hydromorphone, buprenorphine, or oxycodone) for a minimum of 3 days.

#### Study outcome

The median oral morphine equivalent dose in the full population of 2294 patients was 180 mg/24 h. Therefore, patients were divided into two groups: Group 1 requiring less or equal to 180 mg/24 h; group 2 requiring more than 180 mg/24 h.

#### Gene selection and genotyping

In the present study, nineteen SNPs; nine SNPs in the *OPRM1* gene, one in *OPRK1*, three in *OPRD1* and six in *COMT*, were selected as relevant. The SNPs were selected according to previous studies, in which genetic associations to opioid analgesic effects were found [4, 8, 9]. The genotype distributions are listed in Table 1. As minor allele frequency had to be higher than 10% to be included in data analysis, the *OPRK1* was discarded. SNPs were coded to be binary variables for inclusion in the SVM, hence, they were coded so that carriage of the minor allele equals 1 and homozygous for the major allele equals 0 in the model.

**Table 1:** Genotype distribution in the study population

Gene	dbSNP	Genotype	Frequency	Percentage	Cumulative
OPRD1	rs533123	CC	74	3.45	3.45
		CT	695	32.45	35.9
		TT	1373	64.1	100
		Total	2142	100	
	rs678849	CC	480	22.13	22.13
		CT	1009	46.52	68.65
		TT	680	31.35	100
		Total	2169	100	
	rs2236857	AA	676	52.65	52.65
		AG	509	39.64	92.29
		GG	99	7.71	100
		Total	1284	100	
OPRM1	rs1799971	AA	1363	76.44	76.44
		AG	393	22.04	98.48
		GG	27	1.51	99.99
		Total	1783	100	
	rs540825	AA	1234	56.07	56.07
		AT	821	37.3	93.37
		TT	146	6.63	100
		Total	2201	100	
	rs562859	AA	950	43.58	43.58
		AG	962	44.13	87.71
		GG	268	12.29	100
		Total	2180	100	
	rs548646	CC	918	42.68	42.68
		CT	965	44.86	87.54
		TT	268	12.46	100
		Total	2151	100	
	rs1323042	AA	590	27.05	27.05
		AC	1083	49.66	76.71
		CC	508	23.29	100
		Total	2181	100	
	rs618207	CC	956	43.51	43.51
		CT	974	44.33	87.84
		TT	267	12.15	99.99
		Total	2197	100	
	rs639855	GG	1247	56.81	56.81
		GT	806	36.72	93.53
		TT	142	6.47	100
		Total	2195	100	
	rs9479757	AA	20	0.91	0.91
		AG	369	16.8	17.71
		GG	1807	82.29	100
		Total	2196	100	
	rs497976	AA	142	6.49	6.49
		AC	804	36.75	43.24
		CC	1242	56.76	100
		Total	2188	100	
OPRK1	rs7815824	AG	155	7.09	7.09
		GG	2032	92.91	100
		Total	2187	100	
COMT	rs2020917	CC	904	50.31	50.31
		CT	744	41.4	91.71
		TT	149	8.29	100
		Total	1797	100	
	rs5993882	GG	110	5.05	5.05
		GT	793	36.43	41.48
		TT	1274	58.52	100
		Total	2177	100	
	rs4646312	CC	344	15.87	15.87
		CT	1032	47.6	63.47
		TT	792	36.53	100
		Total	2168	100	
	rs165722	CC	413	22.65	22.65
		CT	926	50.8	73.45
		TT	484	26.55	100
		Total	1823	100	
	rs4633	CC	129	21.57	21.57
		CT	307	51.34	72.91
		TT	162	27.09	100
		Total	598	100	
	rs4680	AA	623	27.9	27.9
		AG	1110	49.71	77.61
		GG	500	22.39	100
		Total	2233	100	

*dbSNP* single nucleotide polymorphism database identification, *OPRD* δ-opioid receptor, *OPRM* μ-opioid receptor, *OPRK* κ-opioid receptor, *COMT* catechol-*O*-methyltransferase, *A* Adenine, *G* Guanine; *C* Cytosine, *T* Thymine

#### Machine learning analysis

SVM is a binary classifier, previously used in other prediction studies in pain medicine [10, 11]. In the present study, classification was performed using the libSVM toolbox (version 3.20) for Matlab [11], and a linear kernel function was used to avoid over-fitting of the model [12]. The analysis process is described in details elsewhere [13]. In brief, 10 features along with a label indicating to which opioid dose group the patient belonged to were analyzed in the SVM. The number of features was determined by calculating the accuracy of the classifier by gradually increasing the number of features up to 10. Accuracy was defined as the ratio between correctly classified subjects and total number of subjects in percentage. Based on this, the SVM calculated an optimal decision rule to separate the two groups in the most optimal way. This was done by leave-one-out cross-validation by extracting one patient for testing, and using the remaining patients to train the model. This process was repeated until all patients had been left out. Once a decision rule was determined, a classification accuracy for each of the 10 features was calculated.

#### Statistical analysis

The null hypothesis was that SVM analysis of various SNPs could not predict the required opioid dose in cancer pain patients. All data are reported as mean ± standard deviation. Results from SVM classification were analyzed using Chi square tests. *P* values below 0.05 were considered statistically significant.

### Results

Out of 2294 EPOS participants, 1057 were excluded due to missing one or several of the selected SNPs. The included 1237 patients (637 males and 600 females) had an age of 62.6 ± 12.3 years and BMI of 23.4 ± 4.6). Of these, 662 (53%) required less or equal to 180 mg/24 h oral morphine equivalents, and 575 (47%) required more than 180 mg/24 h.

Machine learning was unable to distinguish between patients requiring less or equal to 180 mg/24 h oral morphine equivalents and those requiring more, using any number of SNP features from 1 to 10. Classification accuracies were: 1 feature; 52.9% (*P* = 0.08), 2 features; 52.9% (*P* = 0.08), 3 features; 52.9% (*P* = 0.08), 4 features; 52.9% (*P* = 0.08), 5 features; 52.9% (*P* = 0.08), 6 features; 53.0% (*P* = 0.07), 7 features; 53.0% (*P* = 0.07), 8 features; 53.0% (*P* = 0.07), 9 features; 53.0% (*P* = 0.07) and 10 features; 52.8% (*P* = 0.08).

### Discussion

This study aimed to investigate whether SVM was able to identify associations between genetic variability and required opioid dose in cancer pain patients. None of the chosen 18 SNPs in the three candidate genes showed significant association with opioid dose, which support earlier findings from the EPOS study in which regular linear regressions were unable to identify correlations [6]. Hence, SVM analysis did not provide additional information regarding prediction of opioid dose using genetic profiling.

## Limitations

A single SNP may only explain a minor part of analgesic variability. A recent study showed that combinations of genetic variants, e.g. in *OPRM1* and *COMT* better explained variability in morphine consumption than single genetic variants [14]. Thus, one advantage of SVM is the possibility to include several SNPs in one analysis, compared to simple linear regression. However, for each SNP three genotypes exist. When using a binary variable which is necessary for SVM analysis, a dominant genetic model is assumed, which may not be optimal. Furthermore, the high number of SNPs included in the analysis may result in a lower accuracy. Additionally, if epistasis, which is the interaction between genes, is present, the effect of one SNP may be altered or masked by the effect of another SNP and thereby reduce the power to detect genetic associations. Thus, from the present study, it cannot be excluded that some SNPs in the selected genes are associated with required opioid dose.

Various statistical methods (based on general linear models) to predict and assess data relationship exist, but here, a SVM approach was selected, according to two reviews [15, 16]. Machine learning differs from conventional statistics, in that there is no predefined model and assumption of data normality, and each patient is classified at the individual level rather than the group. Furthermore, SVM can find non-linear relationships in data, and assess complex associations between several parameters. The latter is different from the traditional one-at-time approach in statistics, where relatively few variables can be tested. On the contrary, the SVM model can be over-fitted to the data and thus loose generalizability. Moreover, the method is relatively new, and many clinicians and researchers are not familiar with the method and output from the model.

Although the method of SVM presents itself with several limitations, methodological limitations of the study design itself may also have had an influence on the result. First, as opioid dose was the primary outcome in the EPOS study, it was used as outcome in the present study as well. Here it was anticipated that opioid dose is related to opioid response, i.e. high dose = less responsiveness to opioid analgesia, and low dose = high responsiveness to opioid analgesia, however this is only a rough estimation and many other factors may be important. Hence, a composite score taking pain intensity, opioid dose and side-effects into consideration might be a better outcome for association analyses of opioid efficacy [17]. However, such a composite score has not been developed or validated for cancer pain patients. In addition, only three opioid receptor genes were included in the analysis. Future studies should include more, as well as SNPs within genes other than those related to opioid receptor signaling, e.g. genes coding for pharmacokinetic factors. For instance, associations between SNPs within the ATP-binding cassette transporter- and cytochrome P450 genes and opioid analgesia have been found in both healthy volunteer and patient studies [4, 17].

Moreover, as human genetic factors only account for part of the inter-individual difference in pain sensitivity, several cofactors may also influence opioid consumption during the post-operative period. These include age, gender, mood, anxiety, drug–drug interactions and epigenetic factors. Thus, human experimental pain studies, conducted in a controlled setting, have shown to be of value to explore the genetic contribution to both pain sensitivity and analgesic responses [18].

## Abbreviations

SNPs: several single nucleotide polymorphisms
EPOS: European Pharmacogenetic Opioid Study
SVM: support vector machine
